# Decoy Receptors Regulation by Resveratrol in Lipopolysaccharide-Activated Microglia

**DOI:** 10.3390/cells12050681

**Published:** 2023-02-21

**Authors:** Rosa Calvello, Chiara Porro, Dario Domenico Lofrumento, Melania Ruggiero, Maria Antonietta Panaro, Antonia Cianciulli

**Affiliations:** 1 Department of Biosciences, Biotechnologies and Environment, University of Bari, I-70125 Bari, Italy; 2 Department of Clinical and Experimental Medicine, University of Foggia, I-71100 Foggia, Italy; 3 Department of Biological and Environmental Sciences and Technologies, Section of Human Anatomy, University of Salento, I-73100 Lecce, Italy

**Keywords:** microglia, resveratrol, decoy receptors, lipopolysaccharide, cytokines

## Abstract

Resveratrol is a polyphenol that acts as antioxidants do, protecting the body against diseases, such as diabetes, cancer, heart disease, and neurodegenerative disorders, such as Alzheimer’s (AD) and Parkinson’s diseases (PD). In the present study, we report that the treatment of activated microglia with resveratrol after prolonged exposure to lipopolysaccharide is not only able to modulate pro-inflammatory responses, but it also up-regulates the expression of decoy receptors, IL-1R2 and ACKR2 (atypical chemokine receptors), also known as negative regulatory receptors, which are able to reduce the functional responses promoting the resolution of inflammation. This result might constitute a hitherto unknown anti-inflammatory mechanism exerted by resveratrol on activated microglia.

## 1. Introduction

Resveratrol (3,5,4′-trihydroxy-trans-stilbene), also called polyphenol, is a stilbenoid belonging to the phytoalexin superfamily, mostly found in red grapes, blueberries, raspberries, mulberries, and peanuts [1]. Resveratrol has two isomers with trans and cis configurations. In this regard, the trans-resveratrol is the non-toxic stereoisomer that has been widely described to have beneficial effects on health [2].

Resveratrol is part of a group of compounds that act as antioxidants do, protecting the body against diseases, such as diabetes, cancer, heart disease, ileitis, obesity, and neurodegenerative disorders, such as Alzheimer’s (AD) and Parkinson’s diseases (PD) [3,4,5,6]. In this respect, in experimental models of both AD and PD, it has been showed that resveratrol exerts neuroprotective actions; however, its application in therapeutic protocols is limited by its poor bioavailability due to quick metabolization in the intestine and liver [3,4,5,6,7].

Resveratrol is able to cross the blood–brain barrier (BBB) via tight junctions, thus carrying out a protective action in the brain tissue that could reduce the loss of neurons, which arises due to neurodegenerative diseases [4,5,6,7,8].

Many studies carried out in recent years have focused on researching the therapeutic potential for the additional treatment of neurodegenerative diseases of many natural compounds, in particular those extracted from plants [5,6,7,8,9,10]. Among the vast diversity of natural compounds that have been studied for their neuroprotective effects, there are polyphenolic compounds, such as curcumin, capsaicin, epigallocatechin gallate, and resveratrol too [7,8,9,11,12,13]. Apart from having antioxidant and anti-inflammatory actions, resveratrol modulates the intracellular signals involved in neurons survival and inhibits beta-amyloid (Aβ) protein aggregation [10,11,12,13,14].

Consistent with these data, it was reported in a mouse model of Parkinson’s-like disease that a resveratrol treatment protects the dopaminergic (DA) neurons of the *Substantia Nigra pars compacta* (SNpc) against neurotoxic insult by modulating inflammatory reactions through SOCS-1 activation [11,12,13,14,15].

Decoy receptors are involved in mechanisms of immune evasion adopted by pathogens, including IL-1R2 and atypical chemokine receptors (ACKRs). In IL-1R2, the lack of the intracellular TIR domain makes this receptor unable to initiate signal transduction following binding with IL-1 [12,16]. The main types of ACKRs are ACKR1, ACKR2, ACKR3, and ACKR4 [13,17]. These molecules are able to recognize and bind specific growth factors or inflammatory cytokines efficiently; however, they are structurally incapable of initiating and transducing signals, acting as a molecular trap for the agonist and for signaling receptor components. All of these members, also referred as chemokine-binding proteins, scavengers, receptor antagonists, negative regulatory receptors, anti-inflammatory ligands, and decoys, act as brakes in the functional responses [14,18].

IL-1R2 functions as a negative regulator of several IL-1 family members, as well as of TLRs, thus it is involved in several pathophysiological contexts in which inflammation and innate and adaptive immune responses play a significative role [19]. ACKR2, previously known as D6, through inhibiting inflammation, mediates the resolution of inflammation in various conditions such as infections, autoimmune diseases, cancer, and neurodegenerative conditions [14,15,16,17,18,19,20].

We reported in a previous work that in LPS-activated cells, the pre-treatment of microglia with resveratrol up-regulated the phosphorylation of JAK1 and STAT3, as well as the expression of the suppressor of cytokine signaling (SOCS)3, demonstrating that the JAK-STAT signaling pathway is involved in the anti-inflammatory effect exerted by resveratrol [15,16,17,18,19,20,21].

The aim of the present research was designed to determine the potential anti-inflammatory effects of resveratrol through the regulation of decoy receptor expression, IL-1R2 and ACKR2, on the activated microglia after prolonged exposure to LPS. The results obtained from this study provide, for the first time, evidence of a new anti-inflammatory mechanism exerted by resveratrol on the activated microglia.

## 2. Materials and Methods

### 2.1. Cell Culture and Treatments

The murine microglial cell line N13 was grown in RPMI 1640 basal medium enriched with 10% heat-inactivated fetal bovine serum (FBS), 1% L-glutamine (2 mM), and 1% penicillin-streptomycin solution (100 U/mL penicillin; 100 μg/mL streptomycin) (Life Technologies-Invitrogen, Milan, Italy) in a CO_2_ incubator set to 5% CO_2_ at 37 °C in a humidified atmosphere until 70% confluence. For the treatments, we used 10 μM resveratrol (trans-3,40, 5-trihydroxystilbene; purity > 99% GC; Sigma Aldrich, St. Louis, MO, USA) and the cell wall component LPS of Salmonella typhimurium at a concentration of 100 ng/mL. N13 cells were submitted to a single treatment with LPS or resveratrol and to a combined treatment with resveratrol, followed up an hour later by LPS (Sigma Aldrich) for 72 h.

### 2.2. Cytotoxicity Assay

Cell viability of N13 cells was evaluated by the MTT (3-(4,5-Dimethylthiazol-2-yl)-2,5-Diphenyltetrazolium Bromide) assay. The cells were seeded in 96-well multi-well plates at a density of 8 × 10^3^/well to be treated with LPS alone or in presence of resveratrol. The MTT was solubilized in PBS 1X to be added to the wells at a working concentration of 0.5 mg/mL starting from a stock solution of 5 mg/mL. After 4 h of incubation in a CO_2_ incubator at 37 °C in a humidified atmosphere, the formazan crystals were solubilized in Dimethyl sulfoxide (DMSO) keeping the plates in agitation for 20 min. Since the amount of formazan is directly proportional to the number of viable cells, it is quantified by measuring the optical density at 560 nm and subtracting the background at 670 nm by using a Victor Multiplate Reader (Wallac, Perkin Elmer, Milan, Italy).

### 2.3. Reverse Transcriptase-Polymerase Chain Reaction (RT-PCR) and End-Point PCR

Cells were harvested, and the total RNA was extracted by using the TRIzol isolation reagent (Invitrogen, Milan, Italy) according to the manufacturer’s instructions. Once isolated, the RNA was reverse transcribed back into cDNA, causing a reaction between 3 μg of total RNA, 40 U of RNase Out (Invitrogen), 40 mU of oligo dT, 0.5 mM dNTP (PCR Nucleotide Mix, Roche Diagnostics, Milan, Italy), and 40 U of Moloney Murine Leukemia Virus Reverse Transcriptase (Roche Diagnostics). The cDNA synthesis was initiated at 37 °C for 59 min and terminated at 95 °C for 5 min to remain at 4 °C. The cDNA was amplified by performing a polymerase chain reaction (PCR) for 30 cycles using a thermal cycler (Eppendorf, Milan, Italy) together with the cDNA of GAPDH, which was used as a reference gene. At the completion of the PCR, TriTrack Loading Dye 6X (Thermo Fisher, Waltham, MA, USA) was added to the amplified samples prior to be loaded onto the agarose gel. The DNA bands were quantified by densitometry with the ImageJ software, and the results were normalized with GAPDH. Primer sequences for the tested genes are reported in Table 1.

### 2.4. Electrophoresis

After 72 h from the treatments, the cells were harvested and lysed with a lysis buffer (1% Triton X-100, 20 mM Tris-HCl, 137 mM NaCl, 10% glycerol, 2 mM EDTA, 1 mM phenylmethylsulfonyl fluoride (PMSF), 20 μM leupeptin hemisulfate salt, and 0.2 U/mL aprotinin) and subjected to many cycles of freezing and thawing to facilitate the lysis. The lysates were obtained by centrifugation at 12,800× *g* for 20 min at 4 °C, and the proteins were quantified by the Bradford’s protein assay [22,23,24,25,26,27,28,29,30,31,32,33,34,35]. A quantity of 25 μg of proteins from each sample were diluted with a sample buffer (0.5 M Tris-HCl pH 6.8, 10% glycerol, 10% (*w*/*v*) SDS, 5% β2-mercaptoethanol, and 0.05% (*w*/*v*) bromophenol blue), and then boiled for 3 min. At the end, the samples were loaded onto 4%–12% SDS precast polyacrylamide gels (BioRad Laboratories, Tokyo, Japan) and fractionated in relation to the size by applying a voltage of 200 V.

### 2.5. Western Blotting

Upon completion of electrophoresis, the resolved proteins were transferred onto ni-trocellulose membranes and blocked with 5% fat-free milk diluted in a solution containing 0.1% (*v*/*v*) Tween 20% and PBS to avoid nonspecific binding. After three ten-minute washes with 0.1% Tween 20-PBS (T-PBS), the membranes were incubated overnight at 4 °C with mouse monoclonal antibody (mAb) anti-CD11b (1:200), anti-iNOS (1:200) mAb, anti-COX-1 (1:200) mAb, anti-COX-2 (1:200) mAb, anti-phospho-cPLA2 (1:200), anti-phospho-IkBα (1:200) mAb, anti-IL-1R2 (1:100) mAb, and anti-ACKR2 receptor (1:100) mAb, and mouse polyclonal Ab anti β-actin (all from Santa Cruz Biotechnology, Inc., Milan, Italy) according to the manufacturer’s protocol. Then, the membranes were washed with 0.1% Tween 20-PBS (for 20 min, 3 times) and incubated with specific horseradish peroxidase (HRP)-conjugated secondary antibody anti-mouse (Santa Cruz Biotechnology, Milan, Italy) diluted to 1:10,000 for 1 h in agitation and in the dark. At the end, the protein bands were highlighted by chemiluminescence, and images were acquired using a ChemiDoc Imaging System. The bands were normalized against β-actin, and the results, expressed as means ± SD, were provide as the relative optical density.

### 2.6. Enzyme-Linked Immunosorbent Assay (ELISA)

The sandwich ELISA was performed following the kit manufacturer’s instructions to measure the levels of TNF-α (Cat. #BMS607-3 Thermo Fisher—Invitrogen Technology, Milan, Italy) and IL-1β (Cat. # BMS6002 Thermo Fisher—Invitrogen Technology, Milan, Italy) cytokines in the cell culture supernatants withdrawn after 72 h from the treatments. Since the intensity of the signal is directly proportional to the concentration of the antigen, the concentration was quantified and expressed in pg/mL. The determinations were performed in triplicate.

### 2.7. NO Production

NO, quantified as the NO^2−^ concentration in the cell culture supernatants, was determined by the Griess assay. The supernatants were collected after 72 h from the treatments and centrifuged to remove possible cellular residues. After adding the Griess Reactive (0.1% N-(1-naphthyl) ethylenediamine dihydrochloride and 1% sulfanilamide in 2.5% H_3_PO_4_) (1:1 *v*/*v*), the samples were incubated in the dark at room temperature for 10 min. At the end, the absorbance was spectrophotometrically measured at 540 nm by using the conditioned medium as a blank to clear the interference of nitrites. The NO^2−^ concentration was calculated by interpolation on a standard curve of sodium nitrite (NaNO_2_) and is expressed as μmol/mL.

### 2.8. PGE_2_ Assay

To measure the PGE_2_ in the cell culture supernatants, we performed the PGE_2_ assay. N13 cells (3 × 10^6^/well) were seeded in 6-well plates, pre-treated with resveratrol for 1 h, and then stimulated with LPS at a concentration of 100 ng/mL. The cultures were maintained at 37 °C for 72 h in a humidified air containing 5% CO_2_. The PGE_2_ levels were determined in the supernatant using the competitive binding immunoassay (Cayman Chemical, Ann Arbor, MI, USA) according to the manufacturer’s instructions. Unstimulated cells were included as a control. The optical density was measured at λ = 405–420 nm using a precision microplate reader, and the PGE_2_ concentration, expressed in ng/mL, was determined by using a PGE_2_ standard curve.

### 2.9. Statistical Analysis

The statistical analysis was carried out with the software package MINITAB Release 14.1 (Minitab Ltd., Coventry, UK). The results were analyzed by the ANOVA one-way followed by the Tukey test, assuming that the *p*-values ≤ 0.05 were significant.

## 3. Results and Discussion

### 3.1. Effects of Resveratrol and Prolonged LPS Treatment on Cell Viability of N13 Microglial Cells

**Figure 1:**
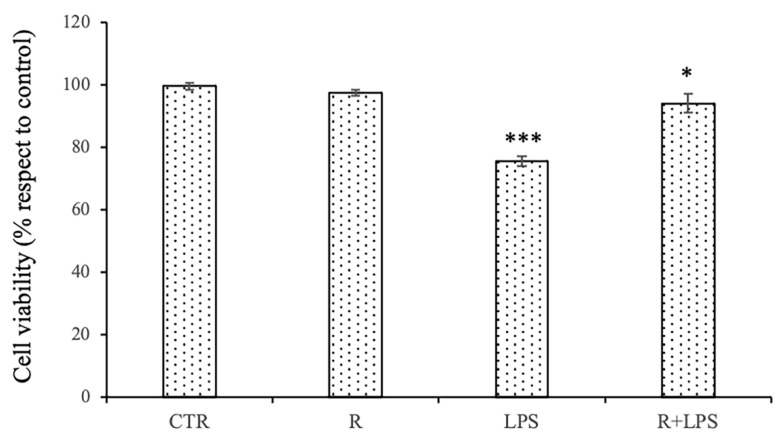
Effects of resveratrol and LPS treatment on cell viability. N13 microglial cells were treated with resveratrol (10 μM) alone or in presence of LPS (100 ng/mL) for 72 h. Cell viability was evaluated by MTT test. Untreated cells used as control (CTR); cells treated with resveratrol (R); cells stimulated with LPS (100 ng/mL) alone (LPS); cells pre-treated with resveratrol before LPS stimulation (R + LPS). Experimental treatments were analyzed in triplicate, and data are expressed as the mean ± SD of five independent experiments. *** *p* < 0.001 LPS vs. CTR; * *p* < 0.05 R + LPS vs. LPS.

The effect of the pre-treatment with resveratrol on the N13 cells treated with LPS was verified by MTT cell viability test. We used an optimal concentration of LPS (100 ng/mL) and an optimal non-toxic resveratrol concentration (10 μM) selected on the basis of the experiments reported in our previous works [15,16,21,23]. Furthermore, in the experiments of this work, we used prolonged exposure to LPS by treating the N13 cells with LPS for 72 h. The viability of the cells exposed for 72 h to 100 ng/mL LPS was significantly reduced in comparison to that of the untreated cells; however, the pre-treatment with resveratrol was able to significantly increase the cell viability in the cells treated with LPS with respect to that of the cells treated with LPS alone (Figure 1).

### 3.2. Resveratrol Modulates CD11b Expression Levels in LPS-Treated N13 Microglial Cells

**Figure 2:**
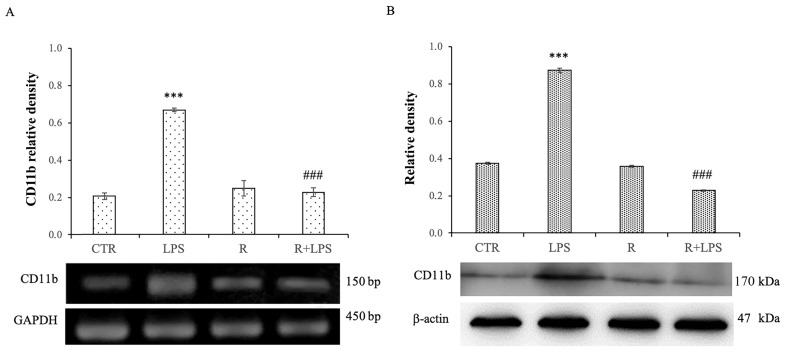
Effects of resveratrol on CD11b expression levels in N13 microglial cells treated with LPS for 72 h. (**A**) PCR analysis of CD11b mRNA expression levels in controls (CTR), N13 cells treated with resveratrol (10 μM) (R), LPS (100 ng/mL) (LPS), LPS, and resveratrol (R + LPS). Results are presented as arbitrary units after normalization against GAPDH, which was used as the resident control. (**B**) Western blotting detection and densitometric analysis of CD11b levels in the microglial cells of control (CTR), LPS (100 ng/mL), resveratrol (10 μM) (R), and resveratrol plus LPS (R + LPS). Protein expression analysis values are expressed as arbitrary units after normalization against β-actin, which was used as a loading control. Data are presented as means ± SD of five independent experiments. *** *p* < 0.001 LPS vs. CTR; ### *p* < 0.001 R + LPS vs. LPS.

The pre-treatment with resveratrol of the N13 cells treated with LPS determined the modulation of the expression of the microglial activation marker CD11b both at transcriptional and post-transcriptional levels (Figure 2). In particular, resveratrol is able to determine a significant decrease in the mRNA expression levels of CD11b in the N13 cells treated with LPS in comparison to those observed in cells treated with LPS alone (Figure 2A). The same results were observed for CD11b protein expression. In this context, the treatment with LPS induced a significantly higher increase in the CD11b protein expression levels in the cells treated with LPS in comparison to that of the control cells. In addition, resveratrol showed the ability to significantly decrease the CD11b protein expression levels in the N13 cells treated with LPS compared to that observed in the cells treated with LPS alone (Figure 2B). These results confirm the role of resveratrol as a modulator of microglial activation even in case of prolonged exposure to LPS.

### 3.3. Effects of Resveratrol on Nitric Oxide Production and Inducible Nitric Oxide Synthase Protein Expression Levels in LPS-Treated N13 Microglial Cells

**Figure 3:**
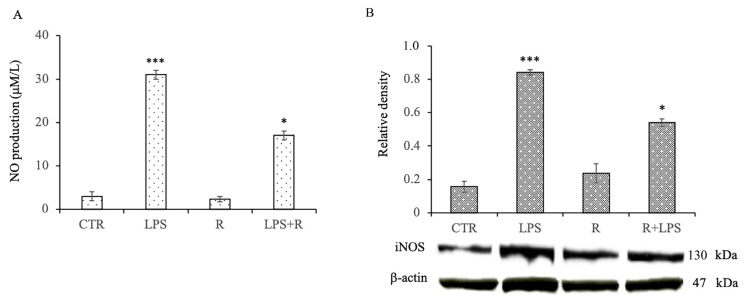
Effects of resveratrol (R) on the cellular oxidative responses in N13 microglial cells stimulated with LPS for 72 h. (**A**) NO release in microglial cells incubated with medium alone (CTR), LPS (100 ng/mL), resveratrol (10 μM) (R), or resveratrol and LPS (R + LPS). The amount of nitrite in the medium was measured by Griess reaction. (**B**) Immunoblotting detection and densitometric analysis of iNOS in microglial incubated with medium alone (CTR), LPS (100 ng/mL), resveratrol (10 μM) (R), or resveratrol and LPS (R + LPS). β-actin was used as a loading control. Results are expressed as means ± SD of five independent experiments. *** *p* < 0.001 LPS vs. CTR.; * *p* < 0.05 R + LPS vs. LPS.

To evaluate the effect of resveratrol on NO production in the N13 cells subjected to prolonged exposure to LPS, the levels of NO produced by the microglia treated for 72 h with LPS in the absence and in the presence of resveratrol were tested. The levels of NO released by the untreated cells and those treated with resveratrol alone were low. The treatment of microglia with LPS for 72 h, on the other hand, resulted in a significant increase in NO release compared to that which was observed in the control cells. Conversely, the cells treated with LPS pre-treated with resveratrol showed a significant reduction of NO production in comparison to that of the cells treated with LPS alone (Figure 3A). In addition, to evaluate whether the inhibitory effect of resveratrol on NO production could derive from an action of resveratrol on the inducible isoform of NO synthase (iNOS), the protein expression of iNOS after the different treatments was determined by the Western blot. Again, significantly higher levels of iNOS protein expression were found in the cells treated with LPS alone in comparison to that which was shown by the untreated cells. Similarly, as observed for NO release, the pre-treatment with resveratrol was able to significantly inhibit the expression of iNOS in the microglia submitted to prolonged exposure to LPS (Figure 3B).

### 3.4. Effects of Resveratrol on Pro-Inflammatory Cytokine Production in LPS-Treated N13 Microglial Cells

**Figure 4:**
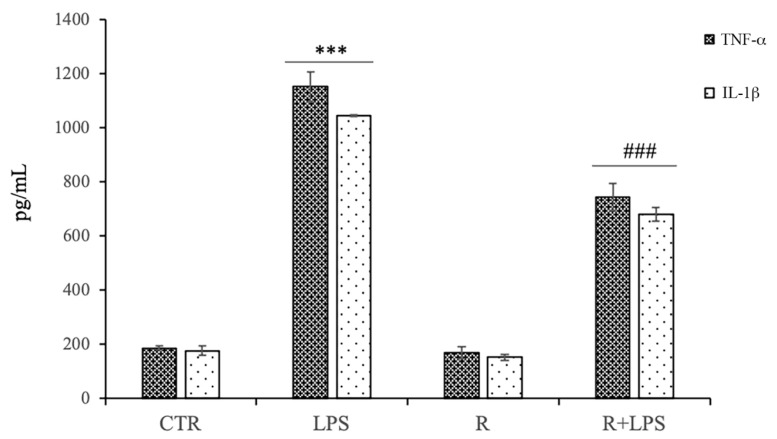
Effects of resveratrol (R) on the pro-inflammatory cytokines release in N13 microglial cells treated with LPS for 72 h. The concentrations of TNF-α and IL-1β measured in supernatants were detected by ELISA and expressed as pg/mL. N13 cells were incubated with medium alone (CTR), LPS (100 ng/mL), resveratrol (10 μM) (R), or resveratrol and LPS (R + LPS). Data are expressed as means ± SD of five independent experiments. *** *p* < 0.001 LPS vs. control; ### *p* < 0.001 R + LPS vs. LPS.

Pro-inflammatory cytokines production levels were assessed in culture supernatants by ELISA both in the presence and absence of resveratrol. As shown in Figure 4, there was a marked increase in TNF-a and IL-1β production in the microglial cells after 72 h of LPS stimulation. No effect by the treatment with resveratrol alone on the pro-inflammatory cytokine production was observed in the microglial cells. Moreover, we observed that the treatment with 10 μg/mL of resveratrol in the LPS-treated cells significantly down-regulated the production levels of pro-inflammatory cytokines in comparison to those of the N13 cells stimulated with LPS alone, suggesting that resveratrol was able to negatively modulate the production levels of pro-inflammatory cytokines in LPS-activated microglial cells.

### 3.5. Effects of Resveratrol on Arachidonic Acid (AA) Pathway in LPS-Treated Microglia

**Figure 5:**
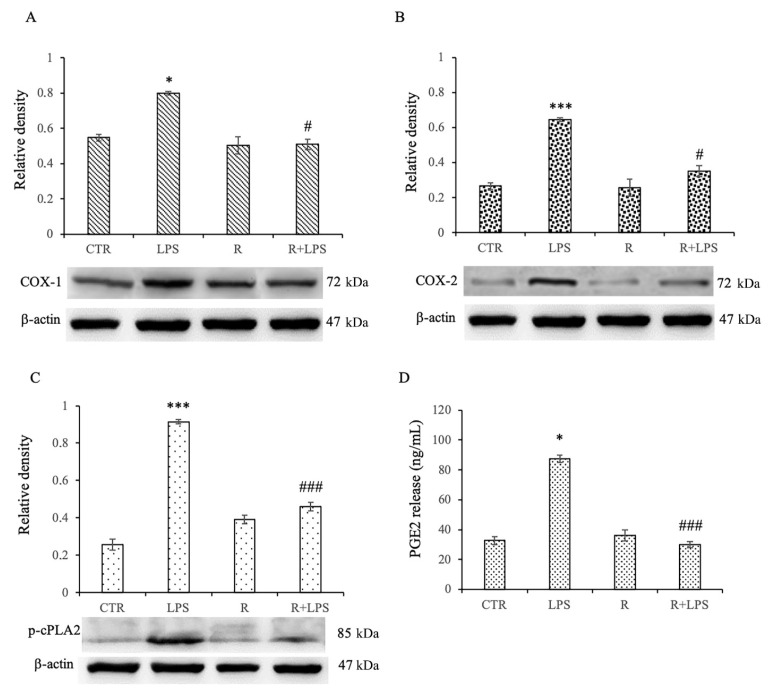
Effects of resveratrol on COX1, COX2, p-cPLA2, and PGE_2_ levels in response to 100 ng/mL LPS in N13 microglial cells. Densitometric analysis and immunoblots of COX-1 (**A**), COX-2 (**B**), and p-cPLA2 (**C**) expression levels in microglial cells. (**D**) PGE_2_ production by N13 cells pre-incubated in medium containing 10 μg/mL of resveratrol for 1 h and then treated with LPS (100 ng/mL) for 72 h. Production of PGE_2_ was detected using an enzyme immunoassay in unstimulated cells (CTR); in microglial cells treated with LPS or resveratrol (R) alone; N13 cells pre-treated with resveratrol before LPS stimulation (R + LPS). Protein expression levels were normalized to β-actin, and values obtained from densitometric analysis are expressed as means ± SD of five independent experiments. * *p* < 0.05 LPS vs. CTR, *** *p* < 0.001 LPS vs. CTR, # *p* < 0.05 R + LPS vs. LPS, ### *p* < 0.001 R + LPS vs. LPS.

Cyclooxygenase-2 (COX-2) and phospholipase A2 (cPLA2) participate in eicosanoid production, such as prostaglandin E2 (PGE_2_), which is implicated in the Arachidonic Acid (AA) pathway and is a key factor in neuroinflammatory and neurodegenerative diseases. Moreover, it is well known that COX-1 could be an important player in neuroinflammation by being predominantly localized in the microglia, and thus, being implicated in the secretion of prostaglandins (PGs) in response to microglia activation [17,24]. For this reason, in microglial cells exposed for a prolonged time to LPS, we have verified the anti-inflammatory ability of resveratrol in terms of the modulation of the of COX-1, COX-2, and p-cPLA2 protein expression. In addition, the evaluation of COX activity by the quantification of the PGE_2_ production by the enzymatic conversion of AA has been widely used and is well accepted as a method to evaluate potential COX inhibitors [18,25]. Therefore, we also verified the inhibitory action of resveratrol on the release of PGE_2_ in N13 cells treated with LPS for 72 h. From our results, it appears to be evident that resveratrol is able to determine a significant decrease in the expression levels of COX-1, COX-2, and p-cPLA2 in the cells treated for 72 h with LPS that had undergone a pre-treatment of 1 h with resveratrol in comparison to those of the cells treated with LPS alone (Figure 5A–C). We observed similar results in the PGE_2_ release assay. Resveratrol, in fact, determined a significant decrease in the release of this inflammatory mediator in the microglia exposed to the prolonged treatment with LPS and pre-treated with resveratrol compared to those subjected to the treatment with LPS alone (Figure 5D). From these results, it is, therefore, possible to highlight that resveratrol, in cases of prolonged inflammation, is able to show an anti-inflammatory effect by inhibiting the AA pathway.

### 3.6. Effects of Resveratrol on NF-kB Pathway in LPS-Treated N13 Microglial Cells

**Figure 6:**
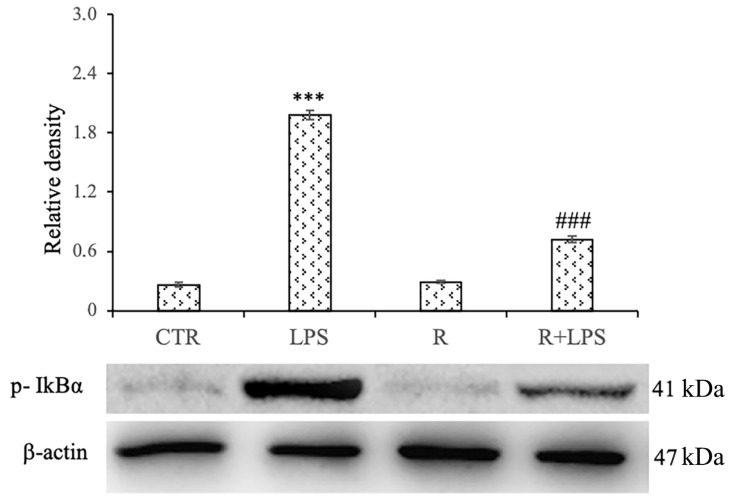
Effects of resveratrol on the LPS-induced phosphorylation of IkB-α in N13 microglial cells treated with LPS for 72 h. Western blotting detection and densitometric analysis of p-IkBα expression levels in the N13 cells of control (CTR), LPS (100 ng/mL), resveratrol (10 μM) (R), and resveratrol plus LPS (R + LPS). For protein expression analysis data are expressed as arbitrary units after normalization against β-actin, which was used as a loading control. *** *p* < 0.001 LPS vs. CTR, ### *p* < 0.001 R + LPS vs. LPS.

In order to evaluate NF-kB activation, we measured the levels of the phosphorylated form of IkBα (p-IkBα), the inhibitory complex of NF-kB, since its phosphorylation is an essential step for NF-kB activation. In this regard, we determined the expression of p-IkB in cell lysates obtained from LPS-stimulated N13 microglial cells. In this context, we observed that the LPS treatment for 72 h significantly increased the expression level of phosphorylated IkB-α protein compared to that of the control cells, and the resveratrol pre-treatment significantly prevented this increase, as revealed by the densitometric analysis (Figure 6). These data indicate that resveratrol inhibited NF-kB activity in the LPS-treated N13 cells by suppressing the degradation of IkB-α, and consequentially, relieving the pro-inflammatory mediator’s expression.

### 3.7. Effects of Resveratrol on IL1-R2 and ACKR2 Decoy Receptor Expression in LPS-Treated Microglia

IL-1R2 is a decoy receptor that causes a block of signal transduction after IL-1 binding. By regulating IL-1R2 expression, cells can modulate inflammation in response to exogenous stimuli. It has been showed that the up-regulation of IL-1R2 in microglial cells and brain endothelial cells attenuates CNS inflammation [12,16].

**Figure 7:**
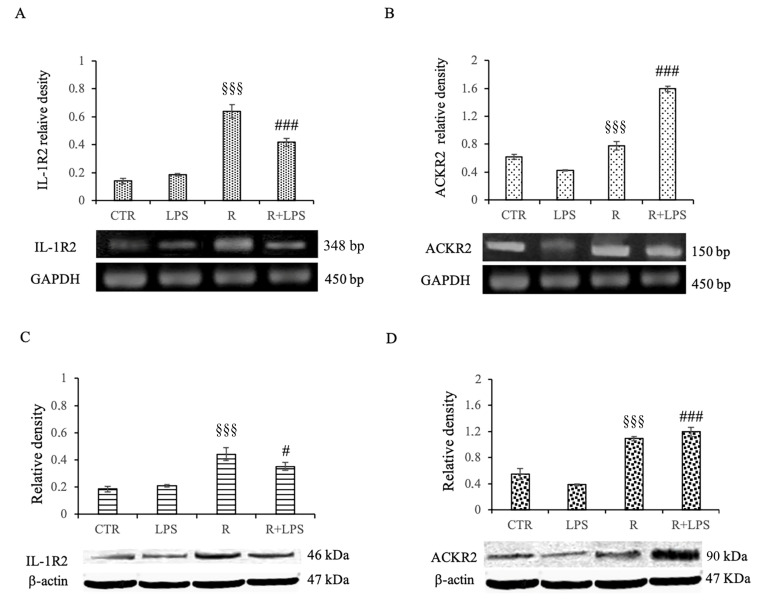
Effects of resveratrol on the decoy receptors mRNA and protein expression levels. N13 microglial cells were pre-incubated in medium containing resveratrol (10 μM) for 1 h, and then treated with LPS (100 ng/mL) for 72 h. PCR analysis of IL-1R2 (**A**) and ACKR2 (**B**) mRNA expression in untreated cells (CTR), N13 cells treated with resveratrol alone (R), LPS alone (LPS), and LPS and resveratrol (R + LPS). Results are presented as arbitrary units after normalization against GAPDH, which was used as the resident control. Western blotting detection and densitometric analysis of IL-1R2 (**C**) and ACKR2 (**D**). For protein expression analysis, the values are expressed as arbitrary units after normalization against β-actin, which was used as a loading control. Results are expressed as means ± SD of five independent experiments. ^§§§^ *p* < 0.001 R vs. LPS; ### *p* < 0.001 R + LPS vs. LPS, # *p* < 0.05 R + LPS vs. LPS.

ACKR2, also known as the D6 decoy receptor, scavenges various inflammatory chemokines, thus affecting the inflammatory microenvironment. In this regard it is thought that the D6 decoy receptor could be a resolving agent in the neuroinflammatory processes because of its capacity to scavenge chemokines, leading to the alleviation of inflammation in different situations, including neuroinflammatory-based neurological disorders [20]. Therefore, in our study, we verified the ability of resveratrol to modulate the expression of decoy receptor IL-1R2 and decoy receptor ACKR2 both in terms of mRNA and protein expression. The analysis of mRNA expression for both the decoy IL1-R2 receptor and the decoy ACKR2 receptor showed a significantly reduced expression of both these receptors in the microglial cells subjected to prolonged exposure to LPS in comparison to that of those cells treated with Resveratrol alone. Interestingly, in the cells exposed to LPS but pre-treated with resveratrol, there was a drastic and highly significant increase in mRNA expression for both of the decoy receptors studied in comparison to that of the cells treated with LPS alone (Figure 7A,B).

These results were confirmed by the Western blotting analysis on IL1-R2 and ACKR2 protein expression. Additionally, in this case, resveratrol was able to cause a significant increase in the protein expression of both IL1-R2 and ACKR2 decoy receptors in the cells treated for 72 h with LPS that received a pre-treatment of 1 h with resveratrol in comparison to that of those cells treated with LPS alone (Figure 7C,D).

All together, these results certainly confirm the already known anti-inflammatory effect that resveratrol elicits on microglial cells in case of neuroinflammation. At the same time, however, these experiments demonstrate, for the first time, the ability of resveratrol to modulate the expression of IL1-R2 and ACKR2 decoy receptors, which could represent a new potential therapeutic target especially in cases of the prolonged inflammation of the CNS.

Based on the previous results evidencing that the resveratrol treatment on the LPS activated microglia responses exerts both an inhibition of pro-inflammatory mechanisms and an induction of anti-inflammatory responses [15,16,21,23], we aimed, in this study, to expand our knowledge regarding the other possible effects of this polyphenolic compound on the inflammatory responses of microglia submitted to a prolonged LPS treatment. Here, we demonstrated that resveratrol, without affecting the viability of these cells, is able to specifically interfere with the pro-inflammatory responses induced by LPS in terms of both the decreased production of IL-1β and the increased production of the IL-1β decoy receptor. IL-1β, a member of the IL-1 family, is a potent pro-inflammatory cytokine in the acute and chronic phases of inflammation, therefore, the reduced production of IL-1β after 72 h of incubation in resveratrol-treated cells demonstrates that this polyphenol could limit the amplification phase of inflammation.

To analyze whether the resveratrol-treated microglia display a reduced ability to react to pro-inflammatory stimuli, we also investigated the response of the cells to LPS in terms of NO and of TNF-a release. In this regard, we detected that after 72 h of treatment, resveratrol was able to significantly reduce the production of both of these mediators. Moreover, we also demonstrated that after a prolonged incubation of microglia cells to LPS, the resveratrol treatment was able to counteract the pro-inflammatory processes down-regulating the IkB degradation, which resulted significantly reduced in comparison to that of the cells treated with LPS alone.

NF-kB is considered to be the most important transcription factor involved in the inflammatory responses, thereby in the regulation of NO, TNF-α, and IL-1β [19,20,26,27].

Previously published papers have reported in other cell types [16,23,28,29] that resveratrol significantly inhibited the degradation of IκBα in microglia stimulated with LPS, as well as the subsequent iNOS expression and production of TNF-α, suggesting that resveratrol can modulate the signaling pathways triggered by pro-inflammatory stimuli, such as LPS. However, in the present study, we observed that this action of resveratrol on the production of TNF-α and the degradation of IκB-α is also evident after a more prolonged incubation time, evidencing how this compound is effective at modulating the inflammatory responses protracted over time and not only in the acute ones.

In addition, we also demonstrated that the resveratrol treatment determined a significant reduction of COX-1, COX-2, and p-cPLA2, which are all mediators of pro-inflammatory responses. Cyclooxygenase exists as COX-1 and COX-2 distinct isoforms [23,24,30,31] and converts arachidonic acid (AA) released by PLA2 acting at the sn-2 position of membrane phospholipids into prostaglandins and other lipid mediators. Both isoforms are important pro-inflammatory enzyme, whose abnormal expression is a significant marker of neuroinflammation, as previously reported [24,31]. Moreover, AA plays also a key role in inflammation and neurodegenerative disorders [25,32]. In mammalians, there are the three major classes of PLA2s, secretory, calcium-independent, and calcium-dependent ones: among them, the calcium-dependent cytosolic PLA2α (cPLA2α) has received the most attention because the cPLA2-AA-COX-2 pathway is an important signaling pathway in different inflammatory paradigms and neurodegeneration [26,33]. In this regard, it has been demonstrated that the oxidative responses observed in many types of brain damage are associated with increased COX activity [27,34]. Moreover, it was reported that a treatment with COX inhibitors may significantly reduce in neuronal and microglial cell LPS- and IL-1β-induced oxidative damage [28,35]. The results of our study are in accordance with ones showing that in mouse microglial cells, the reduction of COX-2 expression observed after a resveratrol treatment could be determined by the inhibition of NF-κB activation [29,36].

Therefore, our data evidence that NF-κB pathway inhibition through the targeting of IκB phosphorylation by resveratrol ultimately may reduce a pro-inflammatory phenotype, thereby down-regulating different mediators, including COX-1, COX-2, and p-cPLA2.

One aspect that is particularly important emerging from our study was the ability of resveratrol to modulate the expression of the so-called decoy receptors, such as IL-1R2 and ACKR2.

IL-1R2, first identified on monocytes, neutrophils, dendritic and B cells, in both human and mice, has been reported to be largely involved in driving myeloid cells polarization, and consequently, orientating the immune response. In fact, anti-inflammatory M2 stimuli, such as IL-4, IL-13, IL-10, IL-27, and aspirin, lead to the up-regulation of IL-1R2 expression, whereas the M1 phenotype activated by pro-inflammatory molecules (such as LPS, IFNγ, and TNF-α) exhibits a down-regulation of IL-1R2 [12,16].

The modulation of IL-1R2 expression has been reported in many cell types as a way to counterbalance and limit sustained inflammation in response to exogenous stimuli. In this regard, IL-1R2 up-regulation in the microglia and brain endothelial cells reduced the brain inflammation in experimental models of IL-1β-induced neurotoxicity, as previously reported [30,31,32,37,38,39].

ACKRs are a group (four in humans) of proteins with a high degree of homology with chemokine receptors. ACKRs are chemotactic receptors; however, since they are devoid of the structural domains required to activate canonical G protein-dependent receptor signaling and chemotactic functions, they do not transduce signals through G proteins and lack chemotactic activity [33,40]. Consequently, ACKRs fail to initiate classical signaling pathways after ligand binding, playing a crucial role as regulatory components of chemokine networks in many physiological and pathological processes.

Interestingly, the resveratrol treatment enhanced the expression of the anti-inflammatory IL-1β decoy receptor IL-1R2 and increased the expression of the other decoy receptor, ACKR2. IL-1R2 is the decoy receptor for IL-1; when IL-1R2 binds to IL-1β, signal transduction cannot be triggered, and consequently, the pro-inflammatory action of this cytokine is neutralized [34,41]. Therefore, the increased expression of IL-1R2 on the microglia surface indicates a reduced responsiveness of these cells to IL-1β stimulation, significantly dampening the pro-inflammatory profile. Moreover, IL-1R2 also exists in soluble form that can be rapidly shed, so the increased release of the soluble form by IL-1R2-overexpressing cells could neutralize the action of IL-1β on other cells, thus reducing the extent of the pro-inflammatory responses.

The results of our pioneering work describe, for the first time, that the resveratrol treatment of the microglia exposed to a prolonged pro-inflammatory stimulus is able to counterbalance inflammatory responses through the regulation of decoy receptors. These findings suggest that the naturally occurring polyphenol resveratrol ability to drive microglial activation, thus regulating the inflammatory response, may help to explain its neuroprotective effects in several in vivo models of neuroinflammation.

## 4. Conclusions

The results of the present in vitro study suggest that polyphenolic compounds, such as resveratrol, may be useful in the treatment of inflammation associated with neurodegeneration and that clinical studies may evaluate the possibility of their use as a therapeutic support strategy. The results of this study highlight the direct effects of resveratrol in the regulation of functional in vitro responses by microglial cells, therefore it would be of considerable importance to investigate the effect of this polyphenol in vivo for future clinical use also in nano-formulations or intranasal spray applications, for example, in order to overcome the bioavailability problems linked to the BBB or metabolism of endothelial cells. In light of these results, we plan to carry out further studies to clarify the modulation mechanisms of the decoy receptors underlying the neuroprotective effects of polyphenols.

## Figures and Tables

**Table 1:** List of primers for RT-PCR in N13 microglial cells.

Gene	Sequence	Sequence References	Product
CD11b forward	5′-GACTCAGTGAGCCCCATCAT-3′	EF101557.1	150 bp
CD11b reverse	5′-AGATCGTCTTGGCAGATGCT-3′		
ACKR2 forward	5′-CCAGCCAAGCCCAGCACGAA-3′	NM_001276719.1	150 bp
ACKR2 reverse	5′-TGCCTCTCACCACCGTCGGT-3′		
IL-1R2 forward	5′-CTCTTGGATAAAGGCATAAGGAATT-3′	NM_001360800.1	348 bp
IL-1R2 reverse	5′-CACACGGCCTCTTGGGTAAGCAGCC-3′		
GADPH forward	5′-ACCACAGTCCATGCCATCAC-3′	NM_001411843.1	450 bp
GADPH reverse	5′-TCCACCACCCTGTTGCTGTA-3′		

## Data Availability

All data presented in this study are available on request from the corresponding author. The data are not uploaded in publicly accessible databases.
